# Association of maternal pre-pregnancy low or increased body mass index with adverse pregnancy outcomes

**DOI:** 10.1038/s41598-021-82064-z

**Published:** 2021-02-15

**Authors:** Jie Tang, Xinhong Zhu, Yanbing Chen, Dongming Huang, Henning Tiemeier, Ruoling Chen, Wei Bao, Qingguo Zhao

**Affiliations:** 1 Department of Preventive Medicine, School of Public Health, Guangzhou Medical University, Room 507, Block 2, Xinzao, Panyu District, 511436 Guangzhou People’s Republic of China; 2 Faculty of Education, Health and Wellbeing, University of Wolverhampton, Millennium City Building, Wulfruna Street, Wolverhampton, WV1 1LY UK; 3 Guangdong Women and Children Hospital, 521-523 Xingnan Street, Panyu District, 511442 Guangzhou People’s Republic of China; 4 Guangdong Institute of Family Planning Science and Technology, 17th Meidong Road, Yuexiu District, 510245 Guangzhou People’s Republic of China; 5 Family Planning Special Hospital of Guangdong, 17th Meidong Road, Yuexiu District, 510245 Guangzhou People’s Republic of China; 6 Department of Child and Adolescent Psychiatry, Erasmus University Medical Centre-Sophia Children’s Hospital, Rotterdam, The Netherlands; 7 Department of Social and Behavioral Sciences, Harvard TH Chan School of Public Health, Boston, USA; 8 Department of Epidemiology, College of Public Health, University of Iowa, Iowa City, IA USA; 9 Key Laboratory of Male Reproduction and Genetics, National Health Committee of China (NHCC, 17th Meidong Road, Yuexiu District, 510245 Guangzhou People’s Republic of China

**Keywords:** Preventive medicine, Nutrition, Preterm birth, Epidemiology, Weight management

## Abstract

This study investigated the association between pre-pregnancy body mass index (BMI) and adverse pregnancy outcomes among women participated in the National Free Preconception Health Examination Project in Guangdong Province, China, and explored these associations according to maternal age. Pre-pregnancy BMI was classified into underweight (BMI < 18.5 kg/m^2^), healthy weight (18.5–23.9 kg/m^2^), overweight (24.0–27.9 kg/m^2^), and obesity (≥ 28.0 kg/m^2^) according to Chinese criteria. Outcomes were preterm birth (PTB, delivery before 37 weeks of gestation), large for gestational age (LGA, birthweight above the 90th percentile for gestational age by infants’ sex), small for gestational age (SGA, birthweight below the 10th percentile for gestational age by infants’ sex), primary caesarean delivery, shoulder dystocia or birth injury, and stillbirth. Adjusted incidence risk ratios (aIRR) were calculated for underweight, overweight and obesity, respectively. Compared with healthy weight, underweight was associated with increased risk of PTB (aIRR 1.06, 95%CI 1.04–1.09) and SGA (1.23, 1.22–1.26) but inversely associated with LGA (0.83, 0.82–0.85), primary caesarean delivery (0.88, 0.87–0.90) and stillbirth (0.73, 0.53–0.99). Overweight was associated with increased risk of LGA (1.17, 1.14–1.19), primary caesarean delivery (1.18, 1.16–1.20) and stillbirth (1.44, 1.03–2.06), but inversely associated with SGA (0.92, 0.90–0.95) and shoulder dystocia or birth injury (0.86, 0.79–0.93). Obesity was associated with increased risk of PTB (1.12, 1.05–1.20), LGA (1.32, 1.27–1.37), primary caesarean delivery (1.45, 1.40–1.50), but inversely associated with SGA (0.92, 0.87–0.97). The aIRRs for underweight, overweight and obesity in relation to these adverse pregnancy outcomes ranged from 0.65 to 1.52 according to maternal age. In Chinese population, maternal pre-pregnancy BMI was significantly associated with the risk of adverse pregnancy outcomes and the risk differs according to maternal age. Further investigation is warranted to determine whether and how counselling and interventions for women with low or increased BMI before pregnancy can reduce the risk of adverse pregnancy outcomes.

## Introduction

Studies have found that maternal low or increased body mass index (BMI) before pregnancy is associated with several adverse pregnancy outcomes including preterm birth (PTB), low or increased birthweight and neonatal mortality^1–4^. However, large cohort studies investigating the association between maternal BMI and adverse pregnancy outcomes have almost always been done in developed countries with high prevalence of overweight and obesity but low prevalence of underweight^5^. There is a shortage of reliable evidence from China or other developing countries where the prevalence of overweight and obesity is increasing but the prevalence of underweight is still high^6^.

Although the causes of adverse pregnancy outcomes are usually unknown, maternal age is the strongest known risk factor. The risk of several adverse pregnancy outcomes (such as PTB and miscarriage) is slightly elevated in the youngest mothers and then rises sharply in older mothers^1,7–9^. However, there are a limited number of studies investigating the association of pre-pregnancy BMI with adverse pregnancy outcomes according to the maternal age, which is vital for risk stratification and making interventions tailored to subgroup population.

We aimed to clarify the association of maternal pre-pregnancy BMI with risk of several adverse pregnancy outcomes in a large population-based cohort study in China, and quantify such risk by maternal age to provide accurate data for risk assessment and counselling in pre-pregnancies.

## Materials and methods

### Study design and participants

We undertook a retrospective cohort study in Guangdong Province, China, from 1st January 2013 to 31st December 2017. Participants were women who participated in the National Free Preconception Health Examination Project (NFPHEP), successfully became pregnant and then had pregnancy outcomes recorded. The NFPHEP covered all rural counties/districts since 2013, with an aim to reduce adverse pregnancy outcomes through providing free health examination before conception and counselling services for reproductive couples. The study design, organization and implementation have been described previously^10–13^.

In the current analysis, we excluded women who did not measure weight or height before pregnancy, who had a chronic disease (including anaemia, hypertension, heart disease, hepatitis B, epilepsy, thyroid disease, chronic nephritis, cancer and diabetes), who had multiple births, and who did not have data on gestational week, birth weight, delivery method, shoulder dystocia or injury birth or stillbirth^13^.

The NFPHEP was approved by the Institutional Review Board of the Chinese Association of Maternal and Child Health Studies. Written informed consent was obtained from the participants before recruitment. The present study was executed jointly by Guangzhou Medical University and Guangdong Institute of Family Planning Science and Technology, in which the review boards determined that this study was exempt for ethical approval owing to the use of de-identified data. All methods of the present study were performed in accordance with the relevant guidelines and regulations.

## Procedure

### Baseline

The NFPHEP was based on the primary health and family planning network. All the reproductive couples who had planned to conceive were recruited. Trained local community health workers collected baseline information, which included demographic characteristics (age, educational level, occupation, ethnicity, migration and address of residence), a history of chronic diseases (anaemia, hypertension, diabetes, heart disease, chronic nephritis, hepatitis B, thyroid disease, cancer and psychiatric diseases), history of pregnancy (gravidity and parity) and history of adverse pregnancy outcomes (preterm birth, miscarriage, abortion, birth defect and stillbirth), lifestyle (maternal active smoking, passive smoking, alcohol consumption and husband smoking). Clinical professionals from the local authorized medical institutions then performed physical examinations. Body weight and height were measured using calibrated instruments with standard measurement procedures^14^.

### Follow up

Participants were followed up by trained local community health workers via telephone every two months to determine whether they had conceived. Local health workers interviewed the women face to face or by telephone within 3 months after conception, documenting their last menstrual period, active smoking, alcohol consumption, and husband smoking during the early stage of the pregnancy. Information regarding where the delivery took place was collected through face to face interview or by telephone within 6 weeks of delivery^13^. Local community health workers then collected data from the medical records at the reference hospital regarding pregnancy outcomes, including gestational age (weeks), birth weight (grams), obstetrical outcomes (caesarean delivery, shoulder dystocia or birth injury), neonatal information (singleton or multiple births and sex) and stillbirth (only collected from 46 counties in 13 cities).

All these baseline data and follow up data were transferred to Guangdong Institute of Family Planning Science and Technology where they were cleaned, complied and de-identified. The endpoint of this study was pregnancy outcomes.

### Categories of pre-pregnancy BMI

Pre-pregnancy BMI was calculated by dividing the weight in kilogram (kg) by the square of the height in meters (m), and was classified into four categories based on the Chinese criteria^15^: underweight (BMI < 18.5 kg/m^2^), healthy weight (18.5–23.9 kg/m^2^), overweight (24.0–27.9 kg/m^2^), and obesity (≥ 28.0 kg/m^2^).

### Outcomes

The NFPHEP obtained pregnancy outcomes from medical records, which were recorded from a gestational age of 28 weeks and 0 days. The outcomes in the present study were PTB (live birth between 28 weeks and 0 days, and 36 weeks and 6 days of gestational age), large for gestational age (LGA, birth weight above the 90th percentile for gestational age by infants’ sex), small for gestational age (SGA, birth weight below the 10th percentile for gestational age by infants’ sex), primary caesarean delivery, shoulder dystocia or birth injury, and stillbirth.

### Statistical analysis

Medians and interquartile range (IRQ) were calculated for age. Means and standard deviations were reported for continuous variables, and frequencies and percentages were reported for categorical variables. Chi-square tests were employed to compare the distribution of BMI categories according to different baseline characteristics. Log-binomial models based on Generalized Estimating Equations (GEE) were employed to estimate the adjusted incidence risk ratios (aIRRs) and 95% CIs of the six outcomes for underweight, overweight and obesity. In each outcome, three models were fitted.

In Model 1, we adjusted for participants’ sociodemographic characteristics, including age at baseline (19–24 years, 25–29 years, 30–34 years, 35–39 years, or 40–50 years), ethnicity (Han or others), educational level (primary school or below, junior high school, senior high school or college or above), occupation (farmer, worker, servicer or others), region (pearl river delta, non-pearl river delta), and migrant population (yes or no). In model 2, we additionally adjusted for history of pregnancy and history of adverse pregnancy outcomes except for primary caesarean delivery^16^, including first pregnancy (yes or no), primipara (yes or no); history of PTB (yes or no), miscarriage (yes or no), induced abortion (yes or no), birth defects (yes or no), or stillbirth (yes or no). In model 3, we additionally adjusted for the lifestyles of the women and the husband, including smoking status of husband before pregnancy and during the early stage of pregnancy (yes or no), smoking and alcohol consumption of women before pregnancy and during the early stage of pregnancy (yes or no), and passive smoking of women before pregnancy (yes or no). Because infant’s sex is associated with all the six outcomes, we adjusted for this variable in all analysis in addition to others listed.

### Sensitivity and subgroup analysis

To examine the robustness of the association of pre-pregnancy BMI with adverse pregnancy outcomes, we performed two other sensitivity analyses with additional adjustment for the length of time from pre-pregnancy examination to the last menstrual period (continuous data) or inclusion of women with self-reported perceived economic pressure (yes or no).

In the subgroup analysis, we divided women into different subgroups on the basis of maternal age. Among these age subgroups, we examined the associations of pre-pregnancy BMI with adverse pregnancy outcomes except for stillbirth. In all the sensitivity and subgroup analysis were adjusted for the aforementioned covariates.

### Missing data

Data were missing in the variables regarding first pregnancy (2910, 0.4%), primipara (2910, 0.4%), active smoke (4960, 0.7%) and husband smoke before pregnancy (23,837, 3.6%), passive smoke (4939, 0.7%), alcohol before pregnancy (6777, 1.07%), active smoke (22,225, 3.3%) and husband smoke during early-stage pregnancy (22,742, 3.4%), alcohol during early-stage pregnancy (22,775, 3.4%). We imputed these missing covariates by using the multiple imputation methodology based on other socio-demographic covariates. The significance level was set at 0.05 and all tests were two-sided. Statistical analyses were conducted using Stata (Version 14.0) and R (version 3.5.2).

## Results

### Participant characteristics

**Figure 1:**
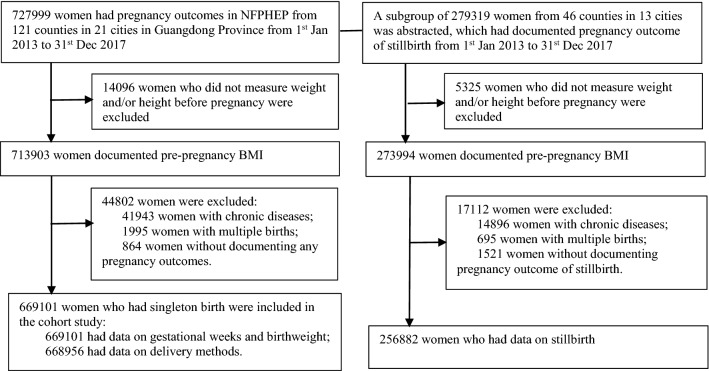
Selection of the study participants.

During the data collection period, 727,999 women had pregnancy outcomes. We excluded 14,096 women who did not measure pre-pregnancy BMI; 41,943 women with chronic diseases; 1995 women with multiple births and 864 women without any data on the gestational age at of delivery, birthweight or delivery method. The remaining 669,101 participants from 121 counties in 21 cities were included in the final analysis to examine the association of pre-pregnancy BMI with PTB, LGA birth and SGA birth. After additional exclusion of 145 women without data on delivery method, 668,956 participants were included in the analysis to examine the association of pre-pregnancy BMI with primary caesarean delivery, shoulder dystocia or birth or birth injury. A subgroup of 256,882 abstracted from 46 counties 13 cities, who had data on stillbirth was included in the analysis to investigate the association of pre-pregnancy BMI with stillbirth. Figure 1 shows the selection of participants for the present study. The sample size and the proportion of the migrant population in each city are in Supplemental Tables 1–3.

Characteristics of the women who had data on gestational age and infants’ birthweight are summarized in Table 1. Overall, the median age was 26 years (IQR 24–29), and 5.1% of the women were older than 35 years. 37.5% of the participants included were from 9 cities in the Pearl River Delta and 10.2% were migrant populations. 45.9% of the participants had an educational level of junior high school or below, 40.3% and 22.3% had an occupation of farmer or worker, 96.4% was of Han nationality, and 65.6% were in their first pregnancy. Among the 669,101 women included, 136,287 (20.3%) were underweight, 69,819 (10.4%) overweight and 14,556 (2.2%) obesity. The distribution of BMI categories with respect to different baseline characteristics were all significantly different (*P* < 0.05). Characteristics of 668,956 women who had data on delivery method and 256,882 women who had data on stillbirth are shown in Supplemental Tables 4 and 5.

**Table 1:** Maternal baseline characteristics according to pre-pregnancy BMI.

	BMI categories*				Total (N = 669,101)
	Underweight (N = 136,287)	Normal (N = 448,439)	Overweight (N = 69,819)	Obesity (N = 14,556)	
**Region (N, %)**					
Non-Pearl river delta	88,056(64.6)	284,469(63.4)	37,907(54.3)	7846(53.9)	418,278(62.5)
Pearl River delta	48,231(35.4)	163,970(36.6)	31,912(45.7)	6710(46.1)	250,823(37.5)
**Migrant population (N, %)**					
Yes	12,634(9.3)	47,452(10.6)	6763(9.7)	1367(9.4)	68,216(10.2)
No	123,653(90.7)	400,987(89.4)	63,056(90.3)	13,189 (90.6)	600,885(89.8)
**Age at baseline (N, %)**					
19–24 years	57,661(42.3)	163,489(36.5)	20,568(29.5)	4185(28.8)	245,903(36.8)
25–29 years	62,836(46.1)	192,562(42.9)	27,301(39.1)	5637(38.8)	288,336(43.1)
30–34 years	12,781(9.4)	65,234(14.6)	14,023(20.1)	3067(21.1)	95,105(14.2)
35–39 years	2710(2.0)	23,373(5.2)	6580(9.4)	1402(9.6)	34,065(5.1)
40–50 years	299(0.2)	3781(0.8)	1347(1.9)	265(1.8)	5692(0.9)
**Education (N, %)**					
Primary school or below	2551(1.9)	9416(2.1)	2043(2.9)	538(3.7)	14,548(2.2)
Junior high school	55,635(40.8)	194,653(43.4)	34,251(49.1)	7551(51.9)	292,090(43.7)
Senior high school	33,403(24.5)	108,876(24.3)	16,054(23.0)	3364(23.1)	161,697(24.2)
College or above	44,698(32.8)	135,494(30.2)	17,471(25.0)	3103(21.3)	200,766(30.1)
**Occupation (N, %)**					
Farmer	52,157(38.3)	179,447(40.0)	31,073(44.5)	6852(47.1)	269,529(40.3)
Worker	30,365(22.3)	100,247(22.4)	15,583(22.3)	3283(22.6)	149,478(22.3)
Servicer	21,044(15.4)	69,247(15.4)	9775(14.0)	1992(13.7)	102,058(15.3)
Others	32,721(24.0)	99,498(22.2)	13,388(19.2)	2429(16.7)	148,036(22.1)
**Ethnicity (N, %)**					
Han	131,068(96.2)	432,388(96.4)	67,372(96.5)	14,024(96.4)	644,852(96.4)
Other	5219(3.8)	16,051(3.6)	2447(3.5)	532(3.7)	24,249(3.6)
**History of pregnancy and adverse pregnancy outcomes (N, %)**					
History of preterm	302 (0.2)	1252(0.3)	302(0.4)	63(0.4)	1919(0.3)
History of miscarriage	3248(2.4)	12,121(2.7)	2563(3.7)	580(4.0)	18,512(2.8)
History of induced abortion	12,926(9.5)	45,913(10.2)	8867(12.7)	1907(13.1)	69,613(10.4)
History of stillbirth	793(0.6)	3424(0.8)	845(1.2)	216(1.5)	5278(0.8)
History of birth defect	216(0.2)	1099(0.3)	286(0.4)	64(0.4)	1665(0.7)
First pregnancy^♯^	101,560(74.5)	296,296(66.1)	34,424(49.3)	6876(52.3)	439,156(65.6)
Primipara^♯^	22,055(16.2)	117,577(26.2)	30,336(43.5)	6538(44.9)	176,506(26.4)
**Lifestyle before pregnancy (N, %)**					
Active smoke^**♯**^	372(0.3)	962(0.2)	207(0.3)	62(0.4)	1603(0.2)
Passive smoke^**♯**^	25,904(19.0)	77,543(17.3)	10,615(15.2)	2081(14.3)	116,143(17.4)
Alcohol^**♯**^	8089(5.9)	28,953(6.5)	3908(5.6)	697(4.8)	41,647(6.2)
Husband smoke^**♯**^	39,916(29.3)	116,958(26.1)	19,819(28.4)	4402(30.2)	181,095(27.1)
**Lifestyle during early pregnancy (N, %)**					
Active smoke^**♯**^	465(0.3)	1438(0.3)	253(0.4)	55(0.4)	2211(0.3)
Alcohol^#^	759(0.6)	2440(0.5)	351(0.5)	73(0.5)	3623(0.5)
Husband smoke^**♯**^	20,422(15.0)	65,014(14.5)	10,545(15.1)	2348(16.1)	98,329(14.7)

^**♯**^Missing data existed.

*The distributions of BMI categories with respect to different baseline characteristics were all statistically (*P* < 0.05), except for ethnicity, smoking, drink during the early stage of pregnancy.

### Association of pre-pregnancy BMI with pregnancy outcomes

Characteristics of the new-borns and frequencies of outcomes with respect to pre-pregnancy BMI are presented in Table 2. Among the 669,101 new-borns included, 33,734 (5.0%) were PTB, 77,204 (11.5%) were LGA births and 64,782 (9.7%) were SGA births. 22,240 (5.0%) healthy weight, 7133 (5.2%) underweight, 3533 (5.1%) overweight and 828 (5.7%) women with obesity had PTB; 52,581 (11.7%) healthy weight, 12,544 (9.2%) underweight, 9783 (14.0%) overweight and 2296 (15.8%) women with obesity had LGA births; 41,711 (9.3%) healthy weight, 16,266 (11.9%) underweight, 5664 (8.1%) overweight and 1141 (7.8%) women with obesity had SGA births.

**Table 2:** Characteristics of the new-borns and frequency of outcomes with respect to pre-pregnancy BMI.

	BMI categories				Total*
	Underweight	Normal	Overweight	Obesity	
**New-born characteristics**^&^					
Gestational week (M ± SD, week)	39.0 ± 1.4	39.0 ± 1.4	39.0 ± 1.5	39.0 ± 1.5	39.0 ± 1.4
Birth weight (M ± SD, week)	3142 ± 136	3187 ± 385	3214 ± 394	3224 ± 412	3181 ± 384
Male sex (n, %)	70,523(51.8)	235,450(52.5)	37,232(53.3)	7621(52.4)	350,826(52.4)
**New-born outcomes**^&^					
Preterm birth	7133(5.2)	22,240(5.0)	3533(5.1)	828(5.7)	33,734(5.0)
Large for gestational age (n, %) ^┼^	12,544(9.2)	52,581(11.7)	9783(14.0)	2296(15.8)	77,204(11.5)
Small for gestational age (n, %)^╪^	16,266(11.9)	41,711(9.3)	5664(8.1)	1141(7.8)	64,782(9.7)
Stillbirth (n, %) ^¶^	50(0.9)	206(1.2)	38(1.8)	10(2.2)	304(1.2)
**Obstetrical outcomes (N, %)** ^Ϯ^					
Caesarean delivery					
Primary	18,742(13.8)	65,454(14.6)	10,264(14.7)	2514(17.3)	96,974(14.5)
Repeat	5125(3.8)	33,969(7.6)	10,471(15.0)	2588(17.8)	52,153(7.8)
Shoulder dystocia or birth injury	1567(1.2)	5199(1.2)	679(1.0)	165(1.1)	7610(1.1)

^┼^Birth weight above the 90th percentile for gestational age by infants’ sex;

^╪^Birth weight below the 10th percentile for gestational age by infants’ sex;

^&^Total number of participants is 669,101;

^¶^Total number of participants is 256,882;

^Ϯ^Total number of participants is 668,956.

Among 668,956 women, there were 96,974 (14.5%) cases of primary caesarean delivery and 7610 cases of shoulder dystocia or birth injury. Primary caesarean delivery occurred in 65,454 (14.6%) healthy weight, 18,742 (13.8%) underweight, 10,264 (14.7%) overweight and 2514 (17.3%) women with obesity women; shoulder dystocia or birth injury occurred in 5199 (1.2%) healthy weight, 1567 (1.2%) underweight, 679(1.1%) overweight and 165 (1.1%) women with obesity.

Among 256,882 deliveries from 46 counties in 13 cities that have recorded stillbirth data, there were 304 (1.2%) stillbirths. Stillbirth occurred in 206 (1.2%) healthy weight women, 50 (0.9%) underweight, 38 (1.8%) overweight and 10 (2.2%) women with obesity.

The aRRs and 95% CIs of the 6 outcomes for pre-pregnancy BMI are shown in Table 3. In the fully adjusted model (model 3), compared with healthy weight, pre-pregnancy underweight was inversely associated with risk of LGA birth (aRR 0.83, 95%CI 0.82–0.85), primary caesarean delivery (0.88, 0.87–0.90) and stillbirth (0.73, 0.53–0.99), but positively associated with risk of PTB (1.06, 1.04–1.09) and SGA (1.23, 1.22–1.26). Pre-pregnancy overweight was inversely associated with risk of SGA birth (0.92, 0.90–0.95) and shoulder dystocia or birth injury (0.86, 0.79–0.93), but positively associated with risk of LGA (1.17, 1.14–1.19), primary caesarean delivery (1.18, 1.16–1.20) and stillbirth (1.44, 1.03–2.06). Pre-pregnancy obesity was inversely associated with risk of SGA birth (0.92, 0.87–0.97) but was positively associated with risk of PTB (1.12, 1.05–1.20), LGA birth (1.32, 1.27–1.37), and primary caesarean delivery (1.45, 1.40–1.50). In all the models related to the 6 outcomes, the aRRs did not substantially change.

**Table 3:** Adjusted risk ratios for adverse pregnancy outcomes according to maternal pre-pregnancy BMI.

Events	Model 1^♯^		Model 2^┼^		Model 3^╪^	
	IRR(95%CI)	*P*	IRR(95%CI)	*P*	IRR(95%CI)	*P*
**Preterm birth (N = 669,101)**						
Normal weight (n = 448,439)	1.00(reference)	…	1.00(reference)	…	1.00(reference)	…
Underweight (n = 136,287)	1.07(1.05–1.10)	< 0.001	1.07(1.04–1.09)	< 0.001	1.06(1.04–1.09)	< 0.001
Overweight (n = 69,819)	1.00(0.97–1.04)	0.847	1.02(0.98–1.05)	0.352	1.02(0.98–1.05)	0.362
Obesity (n = 14,556)	1.14(1.06–1.22)	0.001	1.14(1.06–1.21)	< 0.001	1.12(1.05–1.20)	< 0.001
**Large for gestational age (N = 669,101)**						
Normal weight (n = 448,439)	1.00(reference)	…	1.00(reference)	…	1.00(reference)	…
Underweight (n = 136,287)	0.83(0.81–0.84)	< 0.001	0.83(0.82–0.85)	< 0.001	0.83(0.82–0.85)	< 0.001
Overweight (n = 69,819)	1.18(1.16–1.21)	< 0.001	1.17(1.15–1.19)	< 0.001	1.17(1.14–1.19)	< 0.001
Obesity (n = 14,556)	1.34(1.29–1.39)	< 0.001	1.33(1.27–1.37)	< 0.001	1.32(1.27–1.37)	< 0.001
**Small large for gestational age (n = 669,101)**						
Normal weight (n = 448,439)	1.00(reference)	…	1.00(reference)	…	1.00(reference)	…
Underweight (n = 136,287)	1.25(1.22–1.27)	< 0.001	1.24(1.21–1.26)	< 0.001	1.23(1.22–1.26)	< 0.001
Overweight (n = 69,819)	0.91(0.89–0.93)	< 0.001	0.92(0.90–0.95)	< 0.001	0.92(0.90–0.95)	< 0.001
Obesity (n = 14,556)	0.90(0.86–0.96)	< 0.001	0.92(0.87–0.97)	0.003	0.92(0.87–0.97)	< 0.001
**Primary Caesarean delivery (N = 668,956)**						
Normal weight (n = 448,327)	1.00(reference)		…	…	1.00(reference)	…
Underweight (n = 136,263)	0.89(0.87–0.90)	< 0.001	…	…	0.88(0.87–0.90)	< 0.001
Overweight (n = 69,812)	1.18(1.16–1.20)	< 0.001	…	…	1.18(1.16–1.20)	< 0.001
Obesity (n = 14,554)	1.45(1.40–1.50)	< 0.001	…	…	1.45(1.40–1.50)	< 0.001
**Shoulder dystocia or birth injury (N = 668,956)**						
Normal weight (n = 448,327)	1.00(reference)	…	1.00(reference)	…	1.00(reference)	…
Underweight (n = 136,263)	0.97(0.92–1.03)	0.359	0.97(0.91–1.02)	0.234	0.97(0.92–1.03)	0.309
Overweight (n = 69,812)	0.85(0.78–0.92)	< 0.001	0.86(0.79–0.93)	< 0.001	0.86(0.79–0.93)	< 0.001
Obesity (n = 14,554)	0.99(0.85–1.16)	0.908	1.00(0.86–1.18)	0.914	1.00(0.85–1.16)	0.973
**Stillbirth (N = 256,882)** (n, %)						
Normal weight (n = 173,609)	1.00(reference)		1.00(reference)		1.00(reference)	
Underweight (n = 57,018)	0.74(0.54–1.01)	0.058	0.73(0.54–1.00)	0.049	0.73(0.53–0.99)	0.044
Overweight (n = 21,640)	1.46(1.03–2.07)	0.041	1.45(1.03–2.06)	0.036	1.44(1.03–2.06)	0.033
Obesity (n = 4567)	1.81(0.96–3.43)	0.067	1.80(0.95–3.40)	0.070	1.78(0.94–3.37)	0.075

*IRR* incidence risk ratio.

^♯^Model 1: risk ratios were adjusted for sociodemographic characteristics of maternal (age, education level, occupation, ethnicity, region and migrant population).

^┼^Model 2: risk ratios were additionally adjusted for history of pregnancy (first gestation and primipara) and history of adverse pregnancy outcomes (preterm birth, miscarriage, induced abortion, birth defect, and stillbirth) except for primary caesarean delivery.

^╪^Model 3: risk ratios were adjusted for pre-pregnancy body mass index, active smoking, passive smoking, husband smoking and alcohol consumption status of maternal before pregnancy and active smoking, husband smoking, alcohol drinking status during early stage of pregnancy, in additional to the covariates in Model 2.

### Sensitivity and subgroup analyses

In the sensitivity analyses, the association of pre-pregnancy BMI with the 6 outcomes did not substantially change with additional adjustment for the length of time from pre-pregnancy examination to last menstrual period or inclusion of women self-reported with perceived economic pressure (Supplemental Table 6).

**Figure 2:**
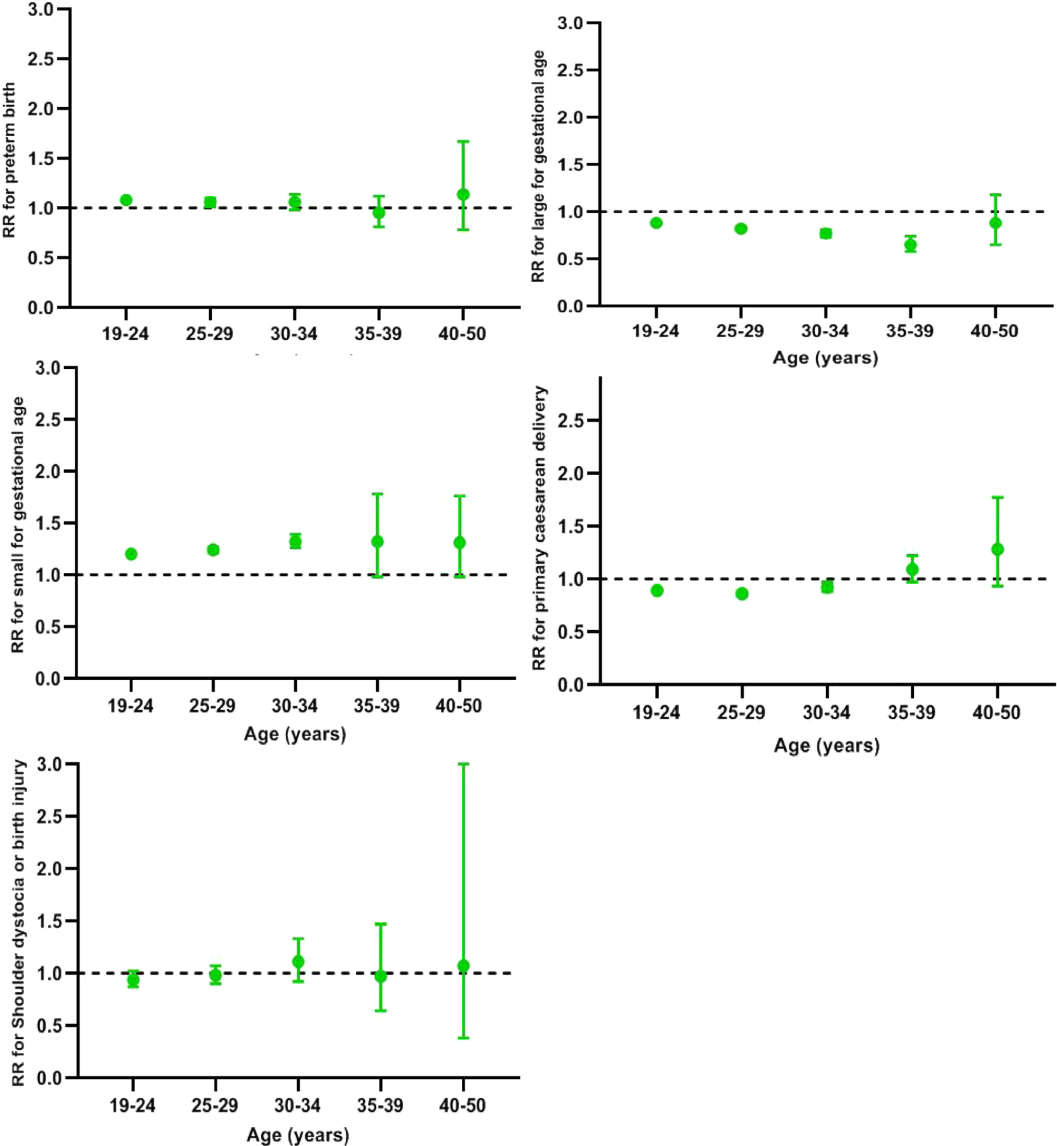
Association of pre-pregnancy underweight with the risks of 5 adverse pregnancy outcomes by maternal age group.

**Figure 3:**
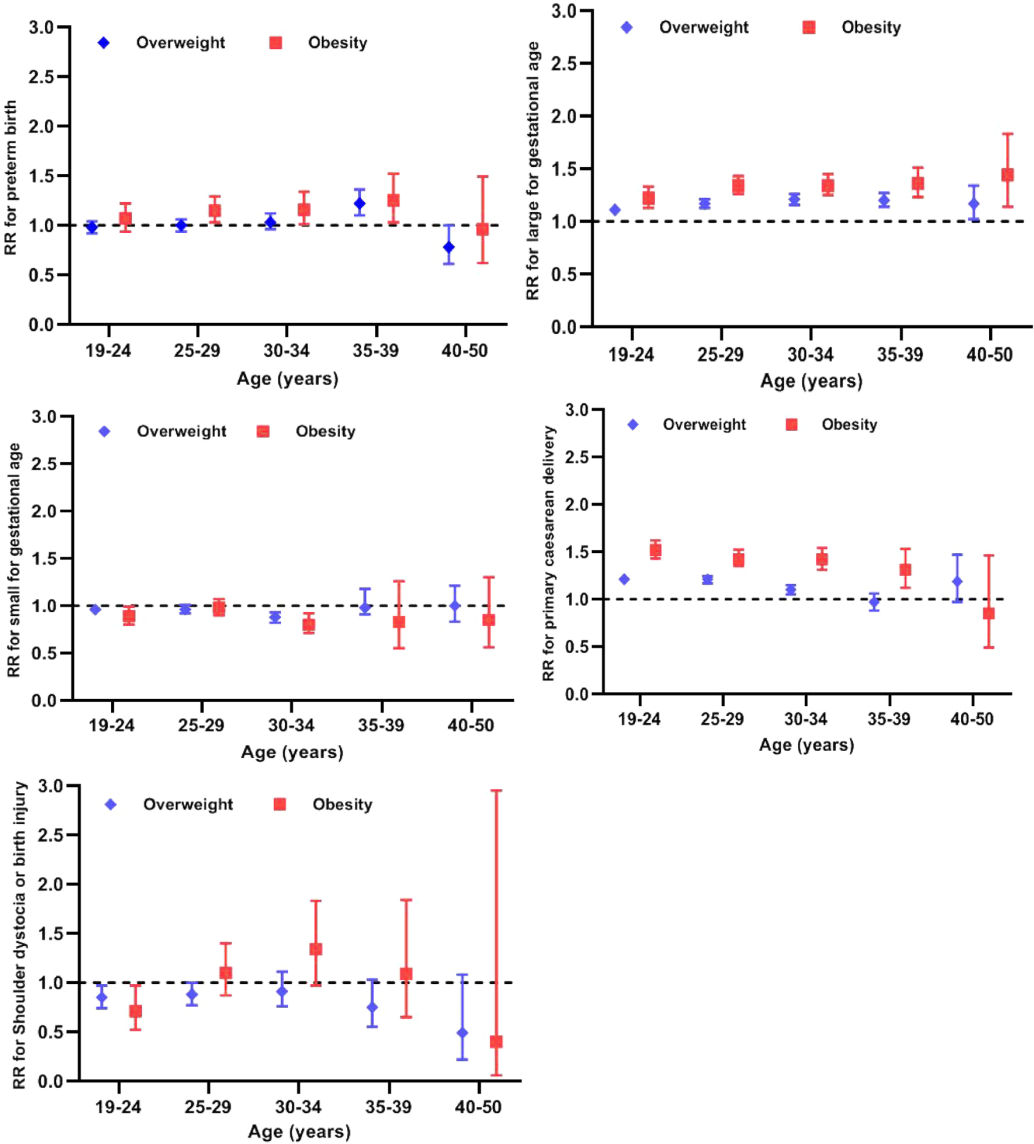
Association of pre-pregnancy overweight and obesity with the risk of 5 adverse pregnancy outcomes by maternal age group.

Subgroup analysis results on the association of pre-pregnancy BMI with risk of the 5 outcomes by maternal age are shown in Figs. 2 and 3. Underweight was inversely associated with risk of LGA birth among those younger than 40 years (0.88, 0.85–0.91; 0.82, 0.80–0.84; 0.77, 0.73–0.81; and 0.65, 0.58–0.74 among those aged 19–24 years, 25–29 years, 30–34 years, 35–39 years, respectively), and primary caesarean delivery among those younger than 35 years (0.89, 0.87–0.92; 0.86, 0.84–0.88; and 0.92, 0.88–0.97 among those aged 19–24 years, 25–29 years and 30–34 years, respectively), but positively associated with risk of SGA among those younger than 35 years (1.2, 1.17–1.23; 1.24, 1.21–1.27; and 1.32, 1.26–1.39 among those aged 19–24 years, 25–29 years and 30–34 years, respectively).

Overweight was inversely associated with risk of SGA birth among those aged 25–29 years (0.92, 0.88–0.96) and 30–34 year (0.88, 0.82–0.92), but positively associated with risk of PTB among those aged 35–39 years (1.22, 1.10–1.36), LGA among all the age groups (19–24 years: 1.11, 1.06–1.16; 1.17, 25–29 years: 1.17, 1.13–1.21; 30–34 years: 1.21, 1.16–1.26; 35–39 years: 1.20, 1.14–1.27 and 40–50 year:1.17, 1.02–1.34), and primary caesarean delivery among those younger than 35 years (19–24 years: 1.21, 1.17–1.25; 25–29 years: 1.21, 1.17–1.24; 30–34 years: 1.10, 1.10–1.15).

Obesity was inversely associated with the risk of SGA among those aged 19–24 years (0.89, 0.80–0.99), but positively associated with risk of PTB among those aged younger than 40 years (25–29 years: 1.15, 1.03–1.29; 30–34 years: 1.16, 1.01–1.34; and 35–39 years: 1.25, 1.03–1.52), LGA among all age groups (19–24 years: 1.22, 1.13–1.33; 25–29 years: 1.34, 1.26–1.43; 30–34 years: 1.34, 1.25–1.45; 35–39 years: 1.36, 1.23–1.51 and 40–50 year:1.44, 1.14–1.83), and primary caesarean delivery among those younger than 40 years (19–24 years: 1.52, 1.43–1.62; 25–29 years: 1.42, 1.35–1.50; 1.42, 30–34 years: 1.31–1.54, and 35–39 years: 1.31, 1.12–1.53).

## Discussion

In this large cohort study conducted in China, we found that compared to women within healthy weight range, those who were underweight had an increased risk of PTB and SGA, but a lower risk of LGA, primary caesarean delivery and stillbirth. Overweight was associated with an increased risk of LGA, primary caesarean delivery and stillbirth, but a lower risk of SGA and shoulder dystocia or birth injury. Obesity was associated with an increased risk of PTB, LGA and primary caesarean delivery, but a lower risk of SGA. Moreover, these associations differed according to maternal age.

Several large, retrospective cohort studies, which were mostly conducted in developed countries, assessed the association of maternal BMI with multiple adverse pregnancy outcomes^3,17^. Sohinee and colleagues^3^ used data from the Aberdeen Maternity and Neonatal Databank (AMND) in the UK, encompassing 24,241 discharges from 1976 to 2015, and found a linear relationship between increased BMI and the risk of developing macrosomia (birth weight over 4000 g), caesarean delivery, while underweight women had better pregnancy outcomes than women with a healthy weight range. Judith and colleagues^17^ analysed singleton pregnancies of 436,414 women in California and found that increased BMI was associated with increased odds ratio of adverse outcomes such as macrosomia and cesarean. Obese women (BMI = 30–39.9) were nearly twice as likely to undergo a caesarean (adjusted OR 1.82, 95%CI: 1.78–1.87) and twice as likely to have an offspring with macrosomia, compared to women within a healthy weight range. However, the association of pre-pregnancy BMI with PTB (< 37 weeks) was only found among underweight women (1.22, 1.16–1.28). Ram and colleagues ^18^ analysed data from the Better Outcomes Registry and Network Ontario, Canada, encompassing 48,780 singleton and 7860 twin births between 2012 and 2016, and found that the risk of caesarean delivery increased with high maternal BMI in both singleton and twin gestations. However, the risk of PTB (< 32 weeks) was only associated with underweight (adjusted RR: 2.10, 95%CI: 1.44–3.08). Of the two studies conducted in China, they used self-reported and recalled pre-pregnancy BMI, or did not adjust for some important confounders including a history of pregnancy and history of adverse pregnancy outcomes, both of which weakened the validity of the association between maternal BMI and pregnancy outcomes^19,20^.

The associations of maternal pre-pregnancy BMI with LGA, SGA and caesarean delivery have been consistent among previous studies both from developed and developing countries^3,17–21^, but not regarding the association of maternal pre-pregnancy BMI with PTB, shoulder dystocia or birth injury and stillbirth. For example, some studies suggested that being underweight was associated with PTB^17,18^, while others indicated that it was obesity^20,21^. Unlike our study, evidence from a recent meta-analysis reported that maternal pre-pregnancy obesity associated with an increased risk of shoulder dystocia (RR: 1.63, 95%CI 1.33–1.99)^22^. Another meta-analysis suggested that pre-pregnancy overweight and obesity were associated with stillbirth (OR, 1.27, 95%CI 1.18–1.36 and 1.81, 95% CI 1.69–1.93, respectively)^23^. However, our study found a significant association between overweight and stillbirth. The discrepancies of the association of pre-pregnancy BMI with adverse pregnancy outcomes may have been related to sample size, methods of research, regions, and the varied characteristics within the study population, such as different prevalence of healthy weight, types and definition of adverse pregnancy outcomes.

Maternal age is the strongest risk factor for adverse pregnancy outcomes^7^. However, there were very few studies that investigated the pre-pregnancy BMI with adverse pregnancy outcomes according to maternal age. The findings of the present study demonstrated that the association of pre-pregnancy with adverse pregnancy outcomes differed according to maternal age, which suggested that the underlying mechanisms causing adverse pregnancy outcomes may differ according to age^1^. A recent study using nationwide birth certificate data from the USA National Vital Statistics System to investigate the association of pre-pregnancy obesity with PTB, also found that the association of obesity with PTB differed according to maternal age^1^. Both studies suggested pre-pregnancy counselling and risk assessment should be stratified by maternal age.

The causes of adverse pregnancy outcomes are complex and multifactorial. However, the associations of pre-pregnancy BMI with pregnancy outcomes could be explained by the uterine environment of the different weight phenotype. Compared with a healthy weight, underweight women have lower plasma volume and rennin-aldosterone response during pregnancy^24^, which may be associated with uteropla-central insufficiency and the increased prevalence of SGA. Previous studies speculated that inflammatory or intrauterine infection might be on the causal pathway between pre-pregnancy underweight or obesity and PTB^25,26^, although the increased prevalence of postpartum infective complications was not observed in some studies^4,25^.

The associations of pre-pregnancy overweight or obesity with adverse pregnancy outcomes may be related to abnormal metabolism of fat. Obese women have higher levels of cord blood tumour necrosis factor α (TNF- α) and RANTES (regulated on activation, normal T cell expressed and secreted upon uptake) during pregnancy, which are known contributors to gestational diabetes mellitus and associated with an increased risk of LGA^27^, whilst LGA was associated with the increased risk of caesarean delivery, shoulder dystocia^28^, and stillbirth^29^. Overweight and obese women have increased insulin resistance in early pregnancy, which manifests clinically in late gestation as glucose intolerance and fetal overgrowth, which also are known risk factors for adverse pregnancy outcomes, such as caesarean delivery, shoulder dystocia and stillbirth^30,31^. Furthermore, women who are overweight or obese are more likely to greater weight gain during pregnancy, which is known risk factors of several pregnancy complications^32^ (such as gestational diabetes mellitus, gestational hypertension) and associated with adverse pregnancy outcomes^33^. Overall, further studies are needed to uncover the potential mechanisms of adverse pregnancy outcomes related to pre-pregnancy BMI.

One of the major strengths of our study is a large sample size. For this cohort, we recruited 669,101 participants and followed up pregnancy outcomes with strict quality controls. The number presented in each category of pre-pregnancy BMI and pregnancy outcomes were enough that multivariable regression models were not over-fitted^13^. The other strength of this study is the high prevalence of underweight in the study population, which has a strong contribution in terms of the relation of underweight and adverse pregnancy outcomes. Additionally, this is the first study examining the association of pre-pregnancy BMI with several adverse pregnancy outcomes according to maternal age, thereby making the results more practical for risk assessment and counselling before pregnancy.

The study has some limitations. Firstly, although all outcomes were abstracted from the medical records, the outcomes are limited to a gestational age of 28 weeks and over. Therefore, we may have underestimated the prevalence of several outcomes including PTB, caesarean delivery, shoulder dystocia or birth injury and stillbirth, and as well as the association between pre-pregnancy BMI and the outcomes^34^. Secondly, the NFPHEP did not measure some important pregnancy and obstetrics complications, such as pregnancy hypertension and pregnancy diabetes. Thus we cannot adjust for these variables, which make the interpretation of our findings difficult^13^. Further studies that include these pregnancy complications for adjustment are warranted to fully understand the association between pre-pregnancy BMI and adverse pregnancy outcomes. Thirdly, we excluded participants with anaemia, hypertension, heart disease, hepatitis B, epilepsy, thyroid disease, chronic nephritis, cancer and diabetes in the present study, thus our findings cannot be generalised to these populations. Fourthly, although we examined the associations of pre-pregnancy BMI with several adverse outcomes according to maternal age, the number of participants who had adverse pregnancy outcomes in 40–50 years group was not enough to calculate the precise aIRRs with precise confidence intervals. Finally, the socio-demographic characteristics, economic, culture, nutritional models and medical service level might not be representative of other countries and regions, suggesting that results from the present study should be verified in different population.

Our findings have important clinical and public health implications. Increased or low pre-pregnancy BMI is common among reproductive age women around the world. Evidence of management of women with different weight in pregnancy were mainly from western countries where have high prevalence of overweight and obesity (including severe obesity) and have different BMI classification^6^, which may not adopt to other developing countries like China. Our findings from more than 660,000 women confirmed that compared with women with healthy weight, a statistically significant increase in risk estimate by 6% of PTB, 23% of SGA in underweight women; 17% of LGA, 18% of primary caesarean delivery, and 44% of stillbirth in overweight women; and 12% of PTB, 32% of LGA, and 45% of primary caesarean delivery in obese women. This suggested that clinical evidence-based recommendation and counselling for management of BMI before and during pregnancy among women of reproductive age might be necessary for reducing the risk of adverse pregnancy outcomes in China. The finding also suggested that the recommendation and counselling should be tailored for different maternal age, as the association of pre-pregnancy BMI with adverse pregnancy differed according to maternal age.

## Conclusion

In conclusion, in this large retrospective cohort study, pre-pregnancy increased or low BMI was significantly associated with the risk of several adverse pregnancy outcomes, and the risk differed according to maternal age. Further investigation is warranted to determine whether and how counselling and interventions for women with increased or low BMI before pregnancy can reduce the risk of adverse pregnancy outcomes, and to understand the underlying mechanisms.

## Supplementary Information

Supplementary Information.

## Data Availability

None of the participant (de-identified) data collected in the study can be shared.
