# Supplementary material for: Association of maternal pre-pregnancy low or increased body mass index with adverse pregnancy outcomes
Source: Sci Rep. 2021 Feb 15;11:3831. doi: 10.1038/s41598-021-82064-z (PMC7884680; doi:10.1038/s41598-021-82064-z)
Supplement: Supplementary file 1 — Supplementary Information. [file 41598_2021_82064_MOESM1_ESM.docx]

**Association of maternal pre-pregnancy low or increased body mass index with adverse pregnancy outcomes**

**Authors:**

Jie Tang, PhD ^1,2,#^; Xinhong Zhu, MD ^3^; Mingzhen Li, MD ^4,5^; Dongming Huang, MD ^4,5^; Henning Tiemeier, Professor ^6, 7^; Ruoling Chen, Professor ^2^; Wei Bao, Assistant Professor ^8^

Qingguo Zhao, Professor^5,6,10,#^

**Authors’ affiliations**

1. Department of Preventive Medicine, School of Public Health, Guangzhou Medical University, Xinzao, Panyu District, 511436, Guangzhou, P.R. China.

2. Faculty of Education, Health and Wellbeing, University of Wolverhampton, Millennium City Building, Wulfruna Street, Wolverhampton, WV1 1LY, UK.

3. Guangdong Women and Children Hospital, 521-523 Xingnan Street, Panyu District, 511442 Guangzhou, P.R. China.

4. Guangdong Institute of Family Planning Science and Technology, 17^th^ Meidong Road, Yuexiu District, 510245 Guangzhou, P.R. China.

5. Family Planning Special Hospital of Guangdong, 17^th^ Meidong Road, Yuexiu District, 510245 Guangzhou, P.R. China.

6. Department of Child and Adolescent Psychiatry, Erasmus University Medical Centre-Sophia Children's Hospital, Rotterdam, the Netherlands.

7. Department of Social and Behavioral Sciences, Harvard TH Chan School of Public Health, Boston, USA.

8. Department of Epidemiology, College of Public Health, University of Iowa, Iowa City, IA, USA

9. National Health Committee of China (NHCC) Key Laboratory of Male Reproduction and Genetics, 17^th^ Meidong Road, Yuexiu District, 510245 Guangzhou, P.R. China.

#, **Corresponding author:** Dr. Jie Tang (Faculty of Education, Health and Wellbeing, University of Wolverhampton, Millennium City Building, Wulfruna Street, Wolverhampton, WV1 1LY, UK, Email: [gytanjie@163.com](mailto:gytanjie@163.com); <Tel:+44> 019023202950) & Professor Qingguo Zhao (Family Planning Special Hospital of Guangdong, 17^th^ Meidong Road, Yuexiu District, 510245 Guangzhou, P.R. China; Email: [zqgfrost@gdszjk.org.cn](mailto:zqgfrost@gdszjk.org.cn); <Tel:+86> 02081375023)

**Appendix Table**

**Supplemental Table 1:** The composition of the study sample having data on gestational and age birthweight (n=669101)

**Supplemental Table 2:** The composition of the study sample having data on delivery methods (n=668956)

**Supplemental Table 3:** The composition of the study sample having data on stillbirth (n=256882)

**Supplemental Table 4:** Maternal baseline characteristics with respect to pre-pregnancy BMI (N=668596)

**Supplemental Table 5:** Maternal baseline characteristics with respect to pre-pregnancy BMI (N=256882)

**Supplemental Table 6:** Additional adjustment for the length of time from pre-pregnancy examination to pregnancy or women self-reported with perceived economic pressure in the multivariable models

**Supplemental Table 1** The composition of the study sample having data on gestational and age birthweight (n=669101)

| **Cities** | **Sample size** | **Proportion** | **Sample size of migrant population** |
| --- | --- | --- | --- |
| Guandzhou | 69571 | 10.4% | 8377 |
| Shenzhen | 42272 | 6.3% | 20846 |
| Foshan | 43121 | 6.4% | 3894 |
| Dongguan | 14979 | 2.3% | 1226 |
| Zhongshan | 24140 | 3.6% | 2749 |
| Zhuhai | 8248 | 1.2% | 1579 |
| Jiangmen | 33792 | 5.1% | 2446 |
| Huizhou | 30143 | 4.5% | 3622 |
| Zhaoqing | 30392 | 4.5% | 2347 |
| Yunfu | 15428 | 2.3% | 1041 |
| Jieyang | 23173 | 3.5% | 774 |
| Meizhou | 29221 | 4.4% | 1507 |
| Shantou | 20107 | 3.0% | 307 |
| Shanwei | 1568 | 0.2% | 54 |
| Heyuan | 8230 | 1.2% | 383 |
| Qingyuan | 24170 | 3.6% | 911 |
| Zhanjiang | 58516 | 8.8% | 2298 |
| Chaozhou | 16897 | 2.5% | 883 |
| Maoming | 132482 | 19.8% | 8672 |
| Yangjiang | 26481 | 4.0% | 1963 |
| Shaoguan | 16170 | 2.4% | 2337 |
| **Total** | **669101** | **100%** | **68216** |

**Supplemental Table 2** The composition of the study sample having data on delivery methods (n=668956)

| **Cities** | **Sample size** | **Proportion** | **Sample size of migrant population** |
| --- | --- | --- | --- |
| Guandzhou | 69484 | 10.4% | 8373 |
| Shenzhen | 42154 | 6.3% | 20841 |
| Foshan | 43101 | 6.4% | 3893 |
| Dongguan | 14979 | 2.2% | 1226 |
| Zhongshan | 24140 | 3.6% | 2749 |
| Zhuhai | 8248 | 1.2% | 1579 |
| Jiangmen | 33792 | 5.1% | 2444 |
| Huizhou | 30122 | 4.5% | 3622 |
| Zhaoqing | 30374 | 4.5% | 2347 |
| Yunfu | 15428 | 2.3% | 1041 |
| Jieyang | 23173 | 3.5% | 774 |
| Meizhou | 29221 | 4.4% | 1507 |
| Shantou | 20107 | 3.0% | 307 |
| Shanwei | 1568 | 0.2% | 54 |
| Heyuan | 8230 | 1.2% | 383 |
| Qingyuan | 24170 | 3.6% | 910 |
| Zhanjiang | 58516 | 8.7% | 2298 |
| Chaozhou | 16897 | 2.5% | 883 |
| Maoming | 132456 | 19.8% | 8672 |
| Yangjiang | 26481 | 4.0% | 1963 |
| Shaoguan | 16170 | 2.4% | 2337 |
| **Total** | **668956** | **100%** | **68203** |

**Supplemental Table 3** The composition of the study sample having data on stillbirth (n=256882)

| **Cities** | **Sample size** | **Proportion** | **Sample size of migrant population** |
| --- | --- | --- | --- |
| Dongguan | 12568 | 5.0% | 1223 |
| Zhongshan | 22896 | 8.9% | 2686 |
| Huizhou | 25773 | 10.0% | 3515 |
| Zhaoqing | 28764 | 11.2% | 2241 |
| Yunfu | 13568 | 5.3% | 1041 |
| Jieyang | 25667 | 10.0% | 765 |
| Meizhou | 23172 | 9.0% | 1507 |
| Shantou | 18077 | 7.0% | 307 |
| Heyuan | 7545 | 2.9% | 372 |
| Qingyuan | 21914 | 8.5% | 904 |
| Chaozhou | 15779 | 6.1% | 883 |
| Yangjiang | 25671 | 10.0% | 1909 |
| Shaoguan | 15488 | 6.0% | 2295 |
| **Total** | **256882** | **100%** | **19648** |

**Supplemental Table 4** Maternal baseline characteristics with respect to pre-pregnancy BMI^*^ (N=668596)

|  | **BMI categories^*^** | | | | **Total**  (N=668956) |
| --- | --- | --- | --- | --- | --- |
|  | Underweight  (N=136263) | Normal  (N=448327) | Overweight  (N=69812) | Obesity  (N=14554) |  |
| **Region (N, %)** | | | |  |  |
| Non-Pearl river delta | 88040(64.6) | 284420(63.4) | 37902(54.3) | 7844(53.9) | 418206(62.5) |
| Pearl River delta | 48223 (35.4) | 163907(36.6) | 31910(45.7) | 6710(46.1) | 250750(37.5) |
| **Migrant population (N, %)** | | | |  |  |
| Yes | 12632(9.3) | 47443(10.6) | 6762(9.7) | 1366(9.4) | 68216(10.2) |
| No | 123631(90.7) | 400884(89.4) | 63050(90.3) | 13188 (90.6) | 600885(89.8) |
| **Age at baseline (N, %)** | | | |  |  |
| 19~24 years | 57652(42.3) | 163459(36.5) | 20567(29.5) | 4183(28.7) | 245852(36.8) |
| 25~29 years | 62823(46.1) | 192511(42.9) | 27298(39.1) | 5637(38.7) | 288269(43.1) |
| 30~34 years | 12780(9.4) | 65216(14.6) | 14021(20.1) | 3067(21.1) | 95084(14.2) |
| 35~39 years | 2709 (2.0) | 23370(5.2) | 6579(9.4) | 1402(9.6) | 34060(5.1) |
| 40~50 years | 299(0.2) | 3780(0.8) | 1347(1.9) | 265(1.8) | 5691(0.9) |
| **Education (N, %)** | | | |  |  |
| Primary school or below | 2551(1.9) | 9413(2.1) | 2043(2.9) | 538(3.7) | 14545(2.2) |
| Junior high school | 55624(40.8) | 194581(43.4) | 34248(49.1) | 7551(51.9) | 292004(43.7) |
| Senior high school | 33399(24.5) | 108860(24.3) | 16053(23.0) | 3362(23.1) | 161674(24.2) |
| College or above | 44689(32.8) | 135473(30.2) | 17468(25.0) | 3103(21.3) | 200733(30.1) |
| **Occupation (N, %)** | | | |  |  |
| Farmer | 52145(38.3) | 179378(40.0) | 31073(44.5) | 6852(47.1) | 269446(40.3) |
| Worker | 30363(22.3) | 100239(22.4) | 15581(22.3) | 3283(22.6) | 149466(22.3) |
| Servicer | 21040(15.4) | 69233(15.4) | 9773(14.0) | 1991(13.7) | 102037(15.3) |
| Others | 32715(24.0) | 99477(22.2) | 13387(19.2) | 2428(16.7) | 148007(22.1) |
| **Ethnicity (N, %)** | | | |  |  |
| Han | 131044(96.2) | 432278(96.4) | 67367(96.5) | 14022(96.3) | 644711(96.4) |
| Other | 5219(3.8) | 16049(3.6) | 2445(3.5) | 532(3.7) | 24245(3.6) |
| **History of pregnancy and adverse pregnancy outcomes (N, %)** | | | |  |  |
| History of preterm | 302 (0.2) | 1252(0.3) | 302(0.4) | 63(0.4) | 1919(0.3) |
| History of miscarriage | 3248(2.4) | 12117(2.7) | 2562(3.7) | 580(4.0) | 18507(2.8) |
| History of induced abortion | 12926(9.5) | 45905(10.2) | 8867(12.7) | 1906(13.1) | 69604(10.4) |
| History of stillbirth | 793(0.6) | 3420(0.8) | 845(1.2) | 216(1.5) | 5274(0.8) |
| History of birth defect | 216(0.2) | 1099(0.3) | 286(0.4) | 64(0.4) | 1665(0.7) |
| First pregnancy^♯^ | 101542(74.5) | 296210(66.1) | 34418(49.3) | 6875(52.3) | 439045(65.6) |
| Primipara^♯^ | 22049(16.2) | 117563(26.2) | 30336(43.5) | 6538(44.9) | 176486(26.4) |
| **Lifestyle before pregnancy (N, %)** | | | |  |  |
| Active smoke**^♯^** | 372(0.3) | 962(0.2) | 207(0.3) | 62(0.4) | 1603(0.2) |
| Passive smoke**^♯^** | 25899(19.0) | 77528(17.3) | 10614(15.2) | 2080(14.3) | 116121(17.4) |
| Alcohol**^♯^** | 8088(5.9) | 28950(6.5) | 3907(5.6) | 697(4.8) | 41642(6.2) |
| Husband smoke**^♯^** | 39911(29.3) | 116937(26.1) | 19818(28.4) | 4401(30.2) | 181067(27.1) |
| **Lifestyle during early pregnancy (N, %)** | | | |  |  |
| Active smoke**^♯^** | 465(0.3) | 1438(0.3) | 253(0.4) | 55(0.4) | 2211(0.3) |
| Alcohol^#^ | 759(0.6) | 2440(0.5) | 351(0.5) | 73(0.5) | 3623(0.5) |
| Husband smoke**^♯^** | 20422(15.0) | 65006(14.5) | 10545(15.1) | 2348(16.1) | 98321(14.7) |

BMI: body mass index.

**^♯^** Denominators provided as missing data existed.

^*^ The distributions of BMI categories with respect to different baseline characteristics were all statistically (*P*<0.05), except for ethnicity, smoking, drink during the early stage of pregnancy

**Supplemental Table 5** Maternal baseline characteristics with respect to pre-pregnancy BMI^*^ (N=256882)

|  | **BMI categories** | | | | **Total**  (N=256882) |
| --- | --- | --- | --- | --- | --- |
|  | Underweight  (N=57018) | Normal  (N=173609) | Overweight  (N=21678) | Obesity  (N=4577) |  |
| **Region (N, %)** | | | |  |  |
| Pearl river delta | 27460(48.2) | 93258 (53.7) | 10646(49.1) | 2088(45.6) | 133452(52.0) |
| Non-Pearl River delta | 29558(51.8) | 80351(46.3) | 11032(50.1) | 2489(54.4) | 123430(48.0) |
| **Migrant population (N, %)** | | | |  |  |
| Yes | 3654(6.4) | 13954(8.0) | 1679(7.7) | 361(7.9) | 19648(7.6) |
| No | 53364(93.6) | 159655(92.0) | 19999(92.3) | 4216 (92.1) | 237234(92.4) |
| **Age at baseline (N, %)** | | | |  |  |
| 19~24 years | 25522(44.8) | 68182(39.3) | 6622(30.5) | 1413(30.9) | 101739(39.6) |
| 25~29 years | 25583(44.9) | 72904(42.0) | 8479(39.1) | 1782(38.9) | 108748(42.3) |
| 30~34 years | 4705(8.3) | 22357(12.9) | 4107(18.9) | 917(20.0) | 32086(12.5) |
| 35~39 years | 1097(1.9) | 8830(5.1) | 2072(9.6) | 397(8.7) | 12396(4.8) |
| 40~50 years | 111(0.2) | 1336(0.8) | 398(1.8) | 68(1.5) | 1913(0.7) |
| **Education (N, %)** | | | |  |  |
| Primary school or below | 1420(2.5) | 4970(2.9) | 970(4.5) | 256(5.6) | 7616(3.0) |
| Junior high school | 21581(37.8) | 65859(37.9) | 8656(39.9) | 1976(43.2) | 98072(38.2) |
| Senior high school | 14473(25.4) | 48335(27.8) | 6018(27.8) | 1237(27.0) | 70063(27.3) |
| College or above | 19544(34.3) | 54445(31.4) | 6034(27.8) | 1108(24.2) | 81131(31.6) |
| **Occupation (N, %)** | | | |  |  |
| Farmer | 20373(35.7) | 60161(34.7) | 7914(36.5) | 1788(39.1) | 90236(35.1) |
| Worker | 14058(24.7) | 45574(26.3) | 5936(27.4) | 1220(26.7) | 66788(26.0) |
| Servicer | 10197(17.9) | 32960(19.0) | 3527(16.2) | 768(16.8) | 47452(18.5) |
| Others | 12390(21.7) | 34914(20.1) | 4301(19.8) | 801(17.5) | 52406(20.4) |
| **Ethnicity (N, %)** | | | |  |  |
| Han | 56763(99.6) | 172711(99.5) | 21565(99.5) | 4551(99.4) | 255590(99.5) |
| Other | 255(0.5) | 898(0.5) | 113(0.5) | 26(0.6) | 1292(0.5) |
| **History of pregnancy and adverse pregnancy outcomes (N, %)** | | | |  |  |
| History of preterm | 114(0.2) | 484(0.3) | 101(0.5) | 26(0.6) | 725(0.3) |
| History of miscarriage | 1116(2.0) | 3778(2.2) | 781(3.6) | 198(4.3) | 5873(2.4) |
| History of induced abortion | 4668(8.2) | 14710(8.5) | 2644(12.3) | 544(11.9) | 22556(8.8) |
| History of birth defect | 67(0.1) | 316(0.2) | 74(0.3) | 19(0.4) | 476(0.2) |
| History of stillbirth | 314(0.6) | 1147(0.7) | 232(1.1) | 63(1.4) | 1756(0.7) |
| First pregnancy^♯^ | 44452(78.0) | 124581(71.8) | 12026(55.5) | 2514(54.9) | 183573(71.5) |
| Primipara^♯^ | 48917(85.8) | 135157(77.9) | 13503(62.3) | 2848(62.2) | 200425(78.0) |
| **Lifestyle before pregnancy (N, %)** | | | |  |  |
| Active smoke**^♯^** | 144(0.3) | 372(0.2) | 77(0.4) | 23(0.5) | 616(0.2) |
| Passive smoke**^♯^** | 13478(23.8) | 38578(22.3) | 5086(23.6) | 969(21.3) | 58111(22.7) |
| Alcohol**^♯^** | 3269(5.8) | 12413(7.2) | 1574(7.3) | 246(5.4) | 17502(6.9) |
| Husband smoke**^♯^** | 17352(31.0) | 45063(26.5) | 7034(33.6) | 1689(38.2) | 71138(28.3) |
| **Lifestyle during early pregnancy (N, %)** | | | |  |  |
| Active smoke**^♯^** | 145(0.3) | 425(0.3) | 56(0.3) | 16(0.3) | 669(0.3) |
| Alcohol^#^ | 270(0.5) | 982(0.6) | 107(0.5) | 26(0.6) | 1385(0.6) |
| Husband smoke**^♯^** | 9765(17.9) | 30092(18.0) | 4244(20.5) | 946(21.8) | 45047(18.3) |

BMI: body mass index.

**^♯^** Denominators provided as missing data existed.

^*^ The distributions of BMI categories with respect to different baseline characteristics were all statistically (*P*<0.05), except for ethnicity, smoking, drink during the early stage of pregnancy

**Supplemental Table 6 Adjusted risk ratios for adverse pregnancy outcomes according to maternal pre-pregnancy BMI**

|  | Model 1^♯^ | |  | Model 2^┼^ | |
| --- | --- | --- | --- | --- | --- |
|  | IRR(95%CI) | *P* |  | IRR(95%CI) | *P* |
| **Preterm birth (N=669101)** | |  |  |  |  |
| Normal weight (n=448439) | 1.00(reference) | … |  | 1.00(reference) | … |
| Underweight (n=136287) | 1.07(1.05-1.10) | <0.001 |  | 1.07(1.04-1.09) | <0.001 |
| Overweight (n=69819) | 1.00(0.97-1.04) | 0.847 |  | 1.02(0.98-1.05) | 0.352 |
| Obesity (n=14556) | 1.14(1.06-1.22) | 0.001 |  | 1.14(1.06-1.21) | <0.001 |
| **Large for gestational age (N=669101)** | |  |  |  |  |
| Normal weight (n=448439) | 1.00(reference) | … |  | 1.00(reference) | … |
| Underweight (n=136287) | 0.83(0.81-0.84) | <0.001 |  | 0.83(0.82-0.85) | <0.001 |
| Overweight (n=69819) | 1.18(1.16-1.21) | <0.001 |  | 1.17(1.15-1.19) | <0.001 |
| Obesity (n=14556) | 1.34(1.29-1.39) | <0.001 |  | 1.33(1.27-1.37) | <0.001 |
| **Small large for gestational age (n=669101)** | |  |  |  |  |
| Normal weight (n=448439) | 1.00(reference) | … |  | 1.00(reference) | … |
| Underweight (n=136287) | 1.25(1.22-1.27) | <0.001 |  | 1.24(1.21-1.26) | <0.001 |
| Overweight (n=69819) | 0.91(0.89-0.93) | <0.001 |  | 0.92(0.90-0.95) | <0.001 |
| Obesity (n=14556) | 0.90(0.86-0.96) | <0.001 |  | 0.92(0.87-0.97) | 0.003 |
| **Primary Caesarean delivery (N=668956)** | |  |  |  |  |
| Normal weight (n=448327) | 1.00(reference) |  |  | … | … |
| Underweight (n=136263) | 0.89(0.87-0.90) | <0.001 |  | … | … |
| Overweight (n=69812) | 1.18(1.16-1.20) | <0.001 |  | … | … |
| Obesity (14554) | 1.45(1.40-1.50) | <0.001 |  | … | … |
| Normal weight (n=448327) | 1.00(reference) | … |  | 1.00(reference) | … |
| Underweight (n=136263) | 0.97(0.92-1.03) | 0.359 |  | 0.97(0.91-1.02) | 0.234 |
| Overweight (n=69812) | 0.85(0.78-0.92) | <0.001 |  | 0.86(0.79-0.93) | <0.001 |
| Obesity (14554) | 0.99(0.85-1.16) | 0.908 |  | 1.00(0.86-1.18) | 0.914 |
| **Stillbirth (N=256882)**(n, ‰) | |  |  |  |  |
| Normal weight (n=173609) | 1.00(reference) |  |  | 1.00(reference) |  |
| Underweight (n=57018) | 0.74(0.54-1.01) | 0.058 |  | 0.73(0.54-1.00) | 0.049 |
| Overweight (n=21640) | 1.46(1.03-2.07) | 0.041 |  | 1.45(1.03-2.06) | 0.036 |
| Obesity (n=4567) | 1.81(0.96-3.43) | 0.067 |  | 1.80(0.95-3.40) | 0.070 |

^♯^: risk ratios were adjusted for sociodemographic characteristics of maternal (age, education level, occupation, ethnicity, region and migrant population), history of pregnancy (first gestation and primipara) and history of adverse pregnancy outcomes (preterm birth, miscarriage, induced abortion, birth defect, and stillbirth) except for primary caesarean delivery, lifestyles (body mass index, active smoking, passive smoking, husband smoking and alcohol consumption status of maternal before pregnancy and active smoking, husband smoking, alcohol drinking status during early stage of pregnancy) and infant sex in both model 1 and 2. Risk ratios were additionally adjusted for the length of time from pre-pregnancy examination to the last menstrual period in model 1; risk ratios were additionally adjusted for self-reported perceived economic pressure in model 2.

2: risk ratios were additionally adjusted

^╪^ Model 3: risk ratios were adjusted for pre-pregnancy, in additional to the covariates in Model 2.
